# Comparison of linkage and association strategies for quantitative traits using the COGA dataset

**DOI:** 10.1186/1471-2156-6-S1-S96

**Published:** 2005-12-30

**Authors:** Matthew B McQueen, Amy Murphy, Peter Kraft, Jessica Su, Ross Lazarus, Nan M Laird, Christoph Lange, Kristel Van Steen

**Affiliations:** 1 Department of Epidemiology, Harvard School of Public Health, 677 Huntington Avenue, Boston, MA 02115, USA; 2 Department of Biostatistics, Harvard School of Public Health, 677 Huntington Avenue, Boston, MA 02115, USA; 3 Channing Laboratory, Harvard Medical School, 181 Longwood Avenue, Boston, MA 02115, USA

## Abstract

Genome scans using dense single-nucleotide polymorphism (SNP) data have recently become a reality. It is thought that the increase in information content for linkage analysis as a result of the denser scans will help refine previously identified linkage regions and possibly identify new regions not identifiable using the sparser, microsatellite scans. In the context of the dense SNP scans, it is also possible to consider association strategies to provide even more information about potential regions of interest. To circumvent the multiple-testing issues inherent in association analysis, we use a recently developed strategy, implemented in PBAT, which screens the data to identify the optimal SNPs for testing, without biasing the nominal significance level. We compare the results from the PBAT analysis to that of quantitative linkage analysis on chromosome 4 using the Collaborative Study on the Genetics of Alcoholism data, as released through Genetic Analysis Workshop 14.

## Background

The rapid advance of genotyping technology has resulted in a wealth of new, high-quality data that may hold promise for the further elucidation of the genetic determinants underlying complex disease. The ultimate utility of such rich data may be limited in scope by existing methods of linkage and association analysis. For example, it is somewhat unclear as to whether increasingly dense single-nucleotide polymorphism (SNP) genome scans will provide the necessary boost in power and/or information to uncover genes of modest effect size. Further, association methods will be subjected to extreme multiple comparison issues, as the number of statistical tests balloon with the vast number of available SNPs. To address the issue of multiple comparisons, recently developed screening tools implemented in PBAT [1] have the potential to be a powerful and unbiased strategy for genome-wide association of family studies [2]. Briefly, the PBAT screening strategy uses the information from uninformative families (information otherwise discarded in a standard family-based association setting) to screen and select the most optimal markers for subsequent testing without biasing the nominal significance level. In this paper, we explore the utility of the PBAT screening method in comparison with quantitative linkage analysis using the Collaborative Study on the Genetics of Alcoholism (COGA) dataset, as released through the Genetic Analysis Workshop 14 (GAW14). We have the unique opportunity to use the same genetic markers for both linkage and association methods, thereby allowing for a more direct and comprehensive comparison of the two strategies.

## Methods

### Description of the dataset

The data provided for Problem 1 in the GAW14 dataset (COGA Study) includes genotypes from the Affymetrix GeneChip™ Human Mapping 10 K array (Affymetrix), comprises 11,555 SNPs as well as quantitative trait information for approximately 1,614 subjects from 143 families of varying size and structure. Here, we focus on the quantitative trait data from the Eyes Closed Resting electroencephalogram experiment, and in particular the measure that corresponds to the first component of a trilinear singular value decomposition of the beta2 band and bipolar electrode data (ECB21). ECB21 was shown to be approximately normally distributed with a mean of 14.53 (standard deviation = 5.5) and ranged from 4.43 to 36.06. There was no substantial skewness or kurtosis found with the ECB21 trait. We restricted our analysis to genotypes from the 786 Affymetrix SNPs on chromosome 4. We chose chromosome 4 because it has been proposed to harbor a region of linkage to the ECB21 phenotype [3-5].

### Quantitative trait linkage analysis

We first performed a multipoint linkage analysis of the ECB21 phenotype using the variance components approach as implemented in MERLIN [6]. Allele frequencies were generated using all genotyped individuals and the marker map provided by Affymetrix was used for the analysis. To assess whether linkage disequilibrium (LD) structure has influence on the linkage signal, we used HAPLOVIEW [7] to provide an indication of LD in the sample. We removed markers that were found to be in strong LD and re-analyzed the sample for linkage.

### Quantitative trait association analysis

Each marker was tested for association with the ECB21 phenotype using the FBAT approach [8] as implemented in PBAT. Association testing was done assuming an additive genetic model and theoretical variance estimate. Through the computer software package PBAT, a new testing strategy has been developed to address the multiple testing issues for family-based association studies [9,10]. The PBAT strategy can be thought of as a screening technique, whereby the most powerful allelic-phenotype association combination is selected from an entire set of allele-phenotype combinations available to the researcher. Unlike standard methods, the PBAT strategy does not bias the nominal significance level of the resulting univariate or multivariate FBAT statistic. PBAT accomplishes this by making use of the *uninformative *families. For example, uninformative families could refer to nuclear families where the two parents are homozygous at a particular locus. The FBAT statistic does not use uninformative families because transmission from a homozygous parent to its offspring is not random [8]. Thus, using the uninformative families to screen for the optimal gene-phenotype combination does not bias the significance level. Specific details about the method can be found in Lange et al. [9,10]. Briefly, the method can be broken down into six steps: 1) Select a subset of phenotypes (or one phenotype) to be tested. 2) Generate a multivariate model that describes the selected phenotype(s) as a function of the genotypes. 3) Replace the observed genotypes for the *informative *families with their expected genotypes conditional on parental genotypes. 4) Estimate the effect-size parameters from the model in step 3. 5) Estimate the power of the selected phenotype-genotype using the conditional power approach [11]. 6) Use the standard univariate FBAT approach on the phenotype-genotype combination that has optimal power from step 5. For the present analysis, we made use of PBAT's screening strategy to select the five most powerful SNPs to be tested and Bonferroni-adjusted the resulting FBAT *p*-values for five tests. The rationale for selecting the five most powerful SNPs was assessed via simulation studies conduced by Van Steen et al. [2] that suggest that this is the optimal strategy in the context of PBAT screening on this scale.

## Results

### Linkage analysis results

**Figure 1:**
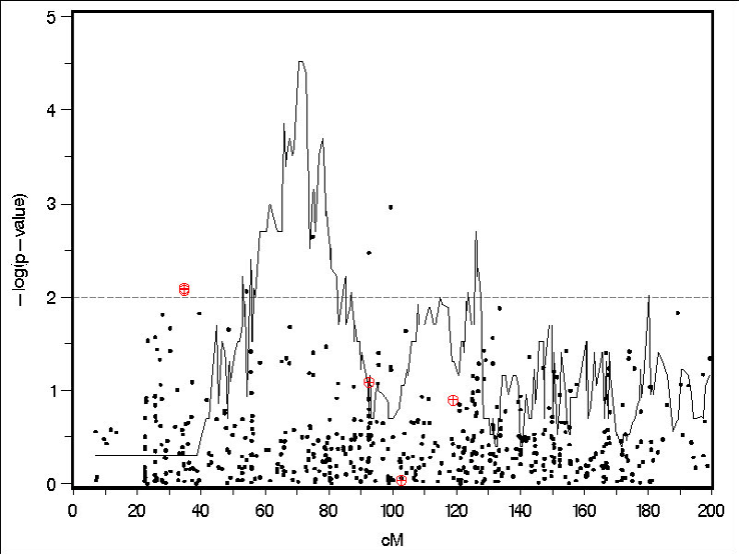
**Linkage and association analysis on chromosome 4**. Graphical representation of analyzing the same markers for linkage and association analysis. The continuous line is a plot of the MERLIN LOD score *p*-value [plotted as -log(*p*-value)] by genetic location (in cM). The red symbols represent the *p*-values [plotted as -log(*p*-value)] of the five SNPs that were found to be most powerful using the PBAT screening strategy. The black dots represent the PBAT *p*-values for all other SNPs tested. The dotted horizontal reference line corresponds to the 5% significance level for the five most powerful PBAT SNPs, Bonferroni-adjusted for five tests (or the 0.01 significance level unadjusted).

Variance component linkage analysis as implemented in MERLIN resulted in a LOD score of 3.55 (*p *= 0.00003) for ECB21 on chromosome 4. The marker corresponding to that LOD score is TSC0149708, located at approximately 72 cM [physical location approximately 57.1 Mbp]. Figure 1 displays the results of the linkage analysis plotted as the -log(*p*-value) using the MERLIN LOD score *p*-value. We sequentially removed markers that were determined by HAPLOVIEW to be in LD both in the linkage region and outside the linkage region and found no difference in the resulting LOD score (data not shown).

### Association analysis results

Table 1 shows the results from the chromosome-wide association analysis using PBAT. Presented in Table 1 are the SNP name, minor allele, allele frequency, number of uninformative families (out of 142), *p*-values, and MERLIN LOD scores for the ten most significant SNPs (lowest *p*-values). The SNP showing the strongest association was TSC0053776 (*p *= 0.0011), located at around 100 cM (physical location approximately 91.9 Mbp). After Bonferroni-adjustment for multiple comparisons (number of tests = 786), none of SNPs achieved statistical significance at the 5% significance level. We also assessed significance using the false discovery rate (FDR) method [12]. Consistent with the Bonferroni-adjustment, one of the SNPs from the FDR analysis reached statistical significance (*q*-value for SNP TSC0053776 = 0.63). Further, the highest LOD score among the 10 most significant SNPs was 2.18, for SNP TSC0286605. This SNP also had the lowest PBAT *p*-value among all SNPs that had LOD scores of greater than 2.0. Other PBAT *p*-values from SNPs with LOD scores over 2.0 were modest, with only three of them having *p*-values of less than 0.05. Using PBAT's screening utility, we focused our analysis on the five most powerful SNPs. Table 2 shows the results of the analysis constrained to these most powerful SNPs and found the top two SNPs to be statistically significant after Bonferroni-adjustment for five tests. The most powerful SNP (TSC0750487) had a p-value of 0.0081. The location of the top two SNPs (TSC0750487 and TSC0568024) are roughly the same in genetic distance at approximately 35 cM, and are physically separated by only 1872 bp. None of the five most powerful SNPs had LOD scores exceeding 1.0. In addition to linkage analysis results, Figure 1 displays the PBAT results as well. There are five red symbols that correspond to the *p*-values for the five most powerful SNPs as identified through PBAT's screening utility (note: two symbols overlap at around 36 cM). The horizontal dotted line refers to the 5% significance level after Bonferroni-adjusting for five comparisons. The black dots are the *p*-values for all other SNPs tested using PBAT.

## Discussion

Using Affymetrix SNPs from the COGA dataset, we identified a region that is linked (LOD = 3.55) to the ECB21 phenotype at approximately 70 cM. This region had been previously identified in the COGA dataset by Reich et al. [3] showing a maximum LOD score of 2.50 using affected (alcoholism diagnosis) sibling pair methodology and microsatellite markers. In addition, we were able to replicate the approximate region of linkage as that found by Porjesz et al. [5] using the same EEG measurement as used for this analysis. Porjesz et al. reported a higher LOD score (over 5.0) than we report here (3.55), however we did not adjust our analysis for age and sex as was done by Porjesz et al.

Using the screening, we also identified two SNPs that are potentially associated with the ECB21 phenotype at approximately 35 cM (*p *= 0.0081 and 0.0085, respectively). As expected, testing each of the 786 SNPs for association resulting in a severe multiple-testing issue, as none of the SNPs across chromosome 4 was found to be statistically significant using either a Bonferroni correction or FDR methods. However, using PBAT's screening strategy allowed us to reduce the number of tests. We chose to test the top five most powerful SNPs as identified by PBAT, and found two SNPs significantly associated with ECB21 at the 5% significance level (after Bonferroni-adjustment for five tests). These two SNPs are physically very close to each other (~2 kb), and when tested together using PBAT's haplotype analysis function, the resulting 2 SNP haplotype maintained its significance and relative power (data not shown).

Interestingly, the selected SNPs were not found to be located directly in the linkage region, as the significant SNPs are approximately 30–40 cM from the maximum LOD score. Furthermore, the LOD scores corresponding to the selected SNPs were 0. The discrepancy in these findings may be explained in a number of ways, particularly when one considers that the alternative hypothesis of the FBAT strategy is the presence of linkage *and *association. First, it is possible, albeit unlikely, that the association approach was able to identify SNPs that are in LD with the linkage region. Second, it has been suggested that association analysis may be more powerful to detect genes of relatively smaller effect sizes [13]. Therefore, it is conceivable that the association strategy identified a novel region that was not detectable by linkage analysis. Third, the association could be completely due to chance, resulting in a false positive for that region. It is also interesting that PBAT did *not *find SNPs that were statistically significant in the linkage region. However, given the alternative hypothesis using the FBAT approach (presence of linkage *and *association) it is possible that the SNPs (in relatively low LD) displaying linkage are *not *associated with the underlying causal locus. It should also be noted that the linkage analysis conducted in the present study was not optimally performed and therefore, we did not maximize the linkage signal. However, the intent of the present study was to compare the two strategies by highlighting key similarities and differences, and not necessarily providing evidence in support of one strategy over the other. Furthermore, we propose that collectively, both strategies may prove useful in high-density genome-wide scans.

## Conclusion

We compared the similarities and differences between linkage analysis and PBAT's approach to association analysis, using the same quantitative trait and using the same marker set. In this brief exploration, we did not find that linkage and association necessarily provided concordant results. Nonetheless, in the context of the high-density SNP scans, we feel that utilizing new strategies for association testing may provide additional information not otherwise discovered using linkage analysis alone.

## Abbreviations

COGA: Collaborative Study on the Genetics of Alcoholism

FDR: False discovery rate

GAW: Genetic Analysis Workshop

LD: Linkage disequilibrium

SNP: Single-nucleotide polymorphism

## Authors' contributions

MBM carried out the linkage analysis and drafted the manuscript. AM carried out portions of the association analysis and aided in drafting the manuscript. PK participated in the design and coordination of the study. JS participated in the design and helped with the analysis. RL participated in the design of the study. NL helped conceive of the study and participated in coordination of the study. CL helped conceive of the study and conducted the association analysis. KVS conceived the study and participated in the coordination of the study.

## Figures and Tables

**Table 1:** Ten SNPs on chromosome 4 with the smallest *p*-values. The ten SNPs that had the smallest *p*-values (unadjusted) associated with ECB21 on chromosome 4 as estimated by PBAT. Corresponding MERLIN LOD scores are also shown.

SNP	Allele	Allele frequency	No. of uninformative families	*p*-value (PBAT)	LOD (MERLIN)
TSC0053776	1	0.17	117	0.0011	0.22
TSC0286605	1	0.38	105	0.0020	2.18
TSC0528073	1	0.29	114	0.0033	0.33
TSC0750487	2	0.33	110	0.0081	0.00
TSC0568024	1	0.33	111	0.0085	0.00
TSC0358705	1	0.25	117	0.0087	0.31
TSC0047225	1	0.37	95	0.0132	0.21
TSC0060984	1	0.30	120	0.0148	0.14
TSC1398038	1	0.24	115	0.0149	0.01
TSC0052464	2	0.46	107	0.0154	0.00

**Table 2:** Five most powerful SNPs on Chromosome 4. The five SNPs with the highest estimated power by PBAT screening on chromosome 4, and their corresponding *p*-values. Corresponding MERLIN LOD scores are also reported.

SNP	Allele	Allele Frequency	No. of Uninformative Families	Power	*p*-value (PBAT)	LOD (MERLIN)
TSC0750487	2	0.33	110	0.96	0.0081	0.00
TSC0568024	1	0.33	111	0.95	0.0085	0.00
TSC0313521	2	0.11	117	0.94	0.1290	0.61
TSC0652507	1	0.34	115	0.94	0.9390	0.40
TSC0528071	1	0.30	115	0.93	0.0828	0.33
